# Limbal epithelial stem cell sheets from young donors have better regenerative potential

**DOI:** 10.1038/s41598-022-17821-9

**Published:** 2022-08-19

**Authors:** Soonwon Yang, Hyun Jung Lee, Soojung Shin, In Yang Park, So-Hyang Chung

**Affiliations:** 1 Department of Ophthalmology and Visual Science, Seoul St. Mary’s Hospital, College of Medicine, The Catholic University of Korea, #222 Banpo-daero, Seocho-gu, Seoul, 06591 Korea; 2 Department of Biochemical Engineering, Seoil University, Seoul, Republic of Korea; 3 Department of Department of Obstetrics and Gynecology, Seoul St. Mary’s Hospital, College of Medicine, The Catholic University of Korea, Seoul, Republic of Korea

**Keywords:** Stem cells, Eye diseases

## Abstract

To investigate the stemness of limbal epithelial stem cell sheets in relation to the donor’s age. Human limbal explants from cadaveric donors were set on human amniotic membrane scaffolds with the xeno-free medium. We evaluated limbal epithelial sheet size, expression of stem/progenitor cell markers, and colony formation efficiency from donors of different age groups (age ≤ 45, age 45–65, and age > 65). Expression of the proliferation marker Ki67, stem/progenitor cell markers p63α and ABCG2, cornea specific marker PANCK, and differentiation marker CK12 were evaluated. To determine the effect of donor age on the storage period of limbal explant sheets, the limbal explant outgrowth sheets were stored in 4 °C for 2 days and analyzed for JC-1, p63α, and PANCK with FACS on each day. From days 6 to 12, the outgrowth area of the limbal epithelial stem cell sheet was significantly larger in the age ≤ 45 groups (296 ± 54.7 mm^2^, day 9) compared to the other two age groups [age 45–65 group (278 ± 62.6 mm^2^), age > 65 group (257 ± 44.0 mm^2^), day 9] (p < 0.01). In terms of stemness, outgrowth cells from aged donors (age > 65) showed lower expression of stem/progenitor cell markers p63α and ABCG2 and decreased CFE compared to the other two groups. There were significantly more p63α+ cells in outgrowth cells in the age ≤ 45 group (18.2 ± 3.6%) compared to the age > 65 group (14.1 ± 4.6%; p < 0.01). Limbal explant outgrowth sheet on the age ≤ 45 group (32.7 ± 7.5%) had higher percentages of cells resisting staining by JC-1 compared with sheets under the age > 65 groups (25.7 ± 7.1%, p < 0.01) (JC-1^low^). Cells from the age ≤ 45 group showed a higher clonogenic capacity than those from the other two age groups (45 < Age ≤ 65 CFE ratio = 0.7 ± 0.16, p < 0.01; 65 < Age CFE ratio = 0.3 ± 0.06, p < 0.01, vs. Age ≤ 45). In the age > 65 group, positive cells of p63α on D0, 1, and 2 were significantly lower compared to those in the age ≤ 45 group on the storage period (p < 0.01, respectively). Our results imply that donors younger than 65 years of age are a better source of limbal epithelial stem cell sheet generation with high regeneration potential.

## Introduction

Limbal stem cells (LSCs) attribute to the maintenance of the corneal epithelium and are endowed with a capacity for self-renewal and extended proliferative potential^1,2^. Limbal stem cell deficiency (LSCD) by multiple etiologies is characterized by impaired corneal wound healing, opaque cornea, conjunctivalization, and partial or total visual loss^3^. In treating LSCD, transplantation of ex vivo limbal epithelial stem cell sheets have achieved an appreciable success rate in terms of ocular surface reconstruction and visual outcomes over the past decade^3–9^. The current treatments involve replenishing the depleted LSCs from a donor source with healthy eyes for the unilateral LSCD patient (autograft) or from a living related or cadaveric donor (allograft) in bilateral cases. During corneal epithelial wound healing, cells displaying stem/precursor cell features undergo a large expansion within the limbal niche and, subsequently, in the cells outgrowing from limbal biopsies set in explant culture^6^. This property is likely to underpin the growth ability of limbal epithelial outgrowth sheets from limbal explants, the most common transplant approach used to restore the health of the eye afflicted by LSCD^7,8^.

The protocol for cultivation of limbal epithelial cells varies greatly from study to study. Each protocol has different culture techniques such as limbal epithelial stem cell sheet generation from limbal explants, isolated epithelial cell cultures, the application of mouse 3T3 feeder cell layer, and different substrates^6,7,10–15^. Our group developed the technique of the cultivation of limbal epithelial sheets on human amniotic membrane scaffolds (HAMS) with a xeno-free medium for clinical applications, which showed enhanced survival of limbal stem/progenitor cells^16^.

Failure of ex vivo limbal epithelial sheet transplantation may be correlated to the depletion of LSCs in expanded culture^17^. Therefore, a generation of limbal epithelial sheets with a high number of stem/progenitor cells from a good donor is critical for long-term regenerative potential in clinical practice. Previous reports showed that the niche of LSC changed with age, with the area of the crypts of the palisades of Vogt being reduced after the age of 65^18^. Therefore, we investigated the stemness of the limbal epithelial stem cell sheets on HAMS according to the donor’s age. We evaluated limbal epithelial sheet size, expression of stem/progenitor cell markers, and colony formation efficiency (CFE) from donors of different age groups (age ≤ 45, age 45–65, and age > 65).

## Results

### Effects of donor’s age on the growth and proliferation of limbal explant outgrowth populations

**Figure 1:**
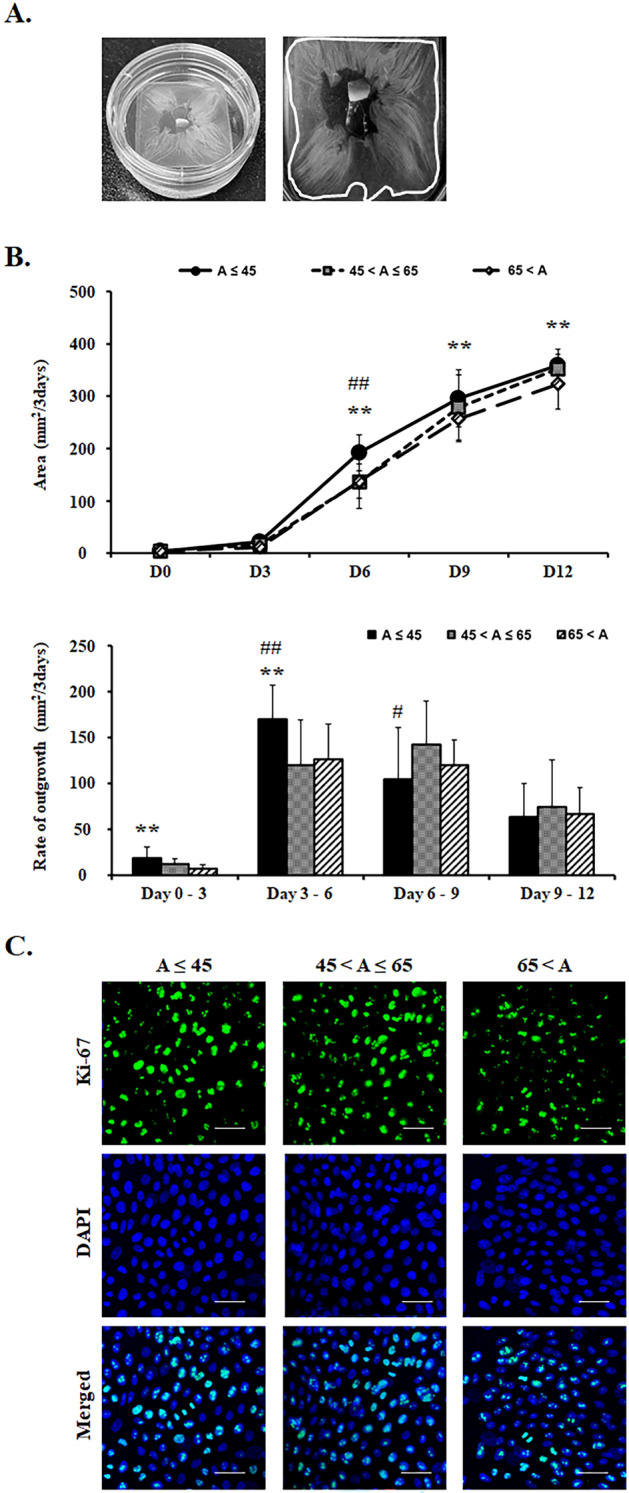
The growth and proliferation of limbal explant outgrowth populations from different donor’s ages. (**A**) Limbal explant outgrowth sheet on human amniotic membrane scaffold. (**B**) Area of limbal explant outgrowth sheets and rate of outgrowth (mm^2^/3 days) by day 12. (**C**) Representative images of explant outgrowths stained for Ki67. Bar = 20 μm. **p < 0.01 (vs. 65 < A), ^#^p < 0.05 (vs. 45 < A ≤ 65), ^##^p < 0.01 (vs. 45 < A ≤ 65), n = 6 from 3 donors.

To investigate the regenerative potential depending on the donor’s age, we compared limbal epithelial stem cell sheets from different donor age groups (age ≤ 45, age 45–65, and age > 65). Limbal explant outgrowth size from age ≤ 45 group demonstrated a conspicuously larger growth pattern compared to the other two age groups by day 6. On day 9, limbal explant outgrowth size from the age ≤ 45 group was 296 ± 54.7 mm^2^, which was significantly larger than the size for the age 45–65 group (278 ± 62.6 mm^2^) or the age > 65 group (257 ± 44.0 mm^2^, p < 0.01) (Fig. 1B). From days 6 to 12, the outgrowth area was markedly larger in the age ≤ 45 group compared to the other two age groups (Fig. 1B, p < 0.01, respectively). In regards to the rate of outgrowth of the sheets observed every 3 days, the age ≤ 45 groups had a higher rate of outgrowth than that of the other two groups from day 3 to day 6 (Fig. 1B). The rate of limbal explant outgrowth in the age ≤ 45 group was 170 ± 36.7 mm^2^, which was significantly higher compared to the age 45–65 group (119 ± 49.2 mm^2^, p < 0.01) or the age > 65 group (126 ± 36.5 mm^2^, p < 0.01) from day 3 to 6. But the rate of outgrowth between the age groups ceased to differ from day 9 to day 12.

To confirm the increased proliferation potential of limbal explant outgrowth sheets on HAMS, Ki67, a proliferation marker, was evaluated. Immunohistochemistry demonstrated that Ki67-positive cells were significantly lower in the age > 65 group than in the other two age groups (p < 0.05, Fig. 1C).

### Effects of donor’s age on the activation of limbal stem/progenitor cells in limbal explant outgrowth sheets

**Figure 2:**
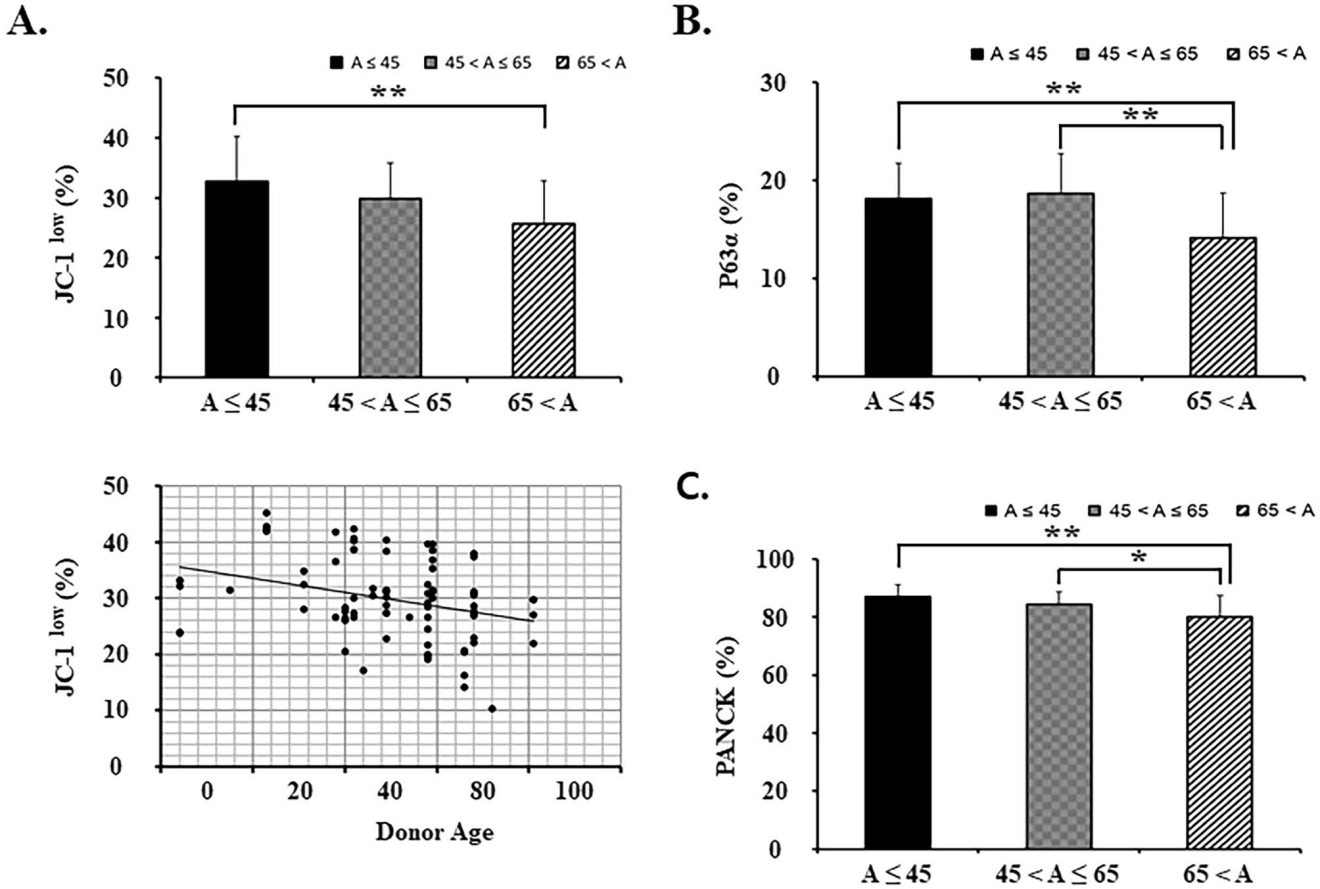
The activation of limbal stem/progenitor cells in limbal explant outgrowth sheets from different donor’s ages. (**A**) Percent of JC-1^low^ cells from FACS analysis and relationship with donor age are shown. JC1 dye exclusion reflects ABCG2 activity and result in the JC1^low^ cohort. (**B**,**C**) p63α and PANCK positive cells from FACS analysis are shown. *p < 0.05 (vs. 65 < A), **p < 0.01 (vs. 65 < A), n = 6 from 3 donors.

To expand the phenotypic characterization of the effect of donor ages, we examined the expression of two markers associated with the limbal stem/progenitor cells, ABCG2 and p63α, and the primary marker of epithelia, PANCK, and corneal epithelial differentiation marker, CK12. Consistent with the concept that JC1^low^ is an ABCG2 substratum, limbal explant outgrowth sheet on the age ≤ 45 group (32.7 ± 7.5%) had higher percentages of cells resisting staining by JC1 compared with sheets under the age > 65 group (25.7 ± 7.1%, p < 0.01) (JC-1^low^)^19^. The percentage of JC-1^low^ cells showed a significant negative correlation with individual donor age (r = − 0.246, p < 0.05, Fig. 2A).

Transcription factor p63, the isoform ΔNp63α, has been linked to stemness and success of early limbal epithelial sheet transplantation. FACS analysis showed significantly more p63α^+^ cells in outgrowth cells in the age ≤ 45 group (18.2 ± 3.6%) compared to the age > 65 group (14.1 ± 4.6%; p < 0.01) but no significant difference from the age 45–65 group (18.7 ± 4.0%). But, the age 45–65 group showed significantly higher positive cells than the age > 65 group (p < 0.01; Fig. 2B). FACS analysis of epithelial cell marker PANCK demonstrated a notably higher number of positive cells in the age ≤ 45 group (87.1 ± 4.2%) compared to the age > 65 group (80.0 ± 7.4%, p < 0.01). Moreover, it was seen that the age 45–65 group (84.3 ± 4.3%) showed a significantly higher number of positive cells than the age > 65 group (p < 0.05; Fig. 2C).

**Figure 3:**
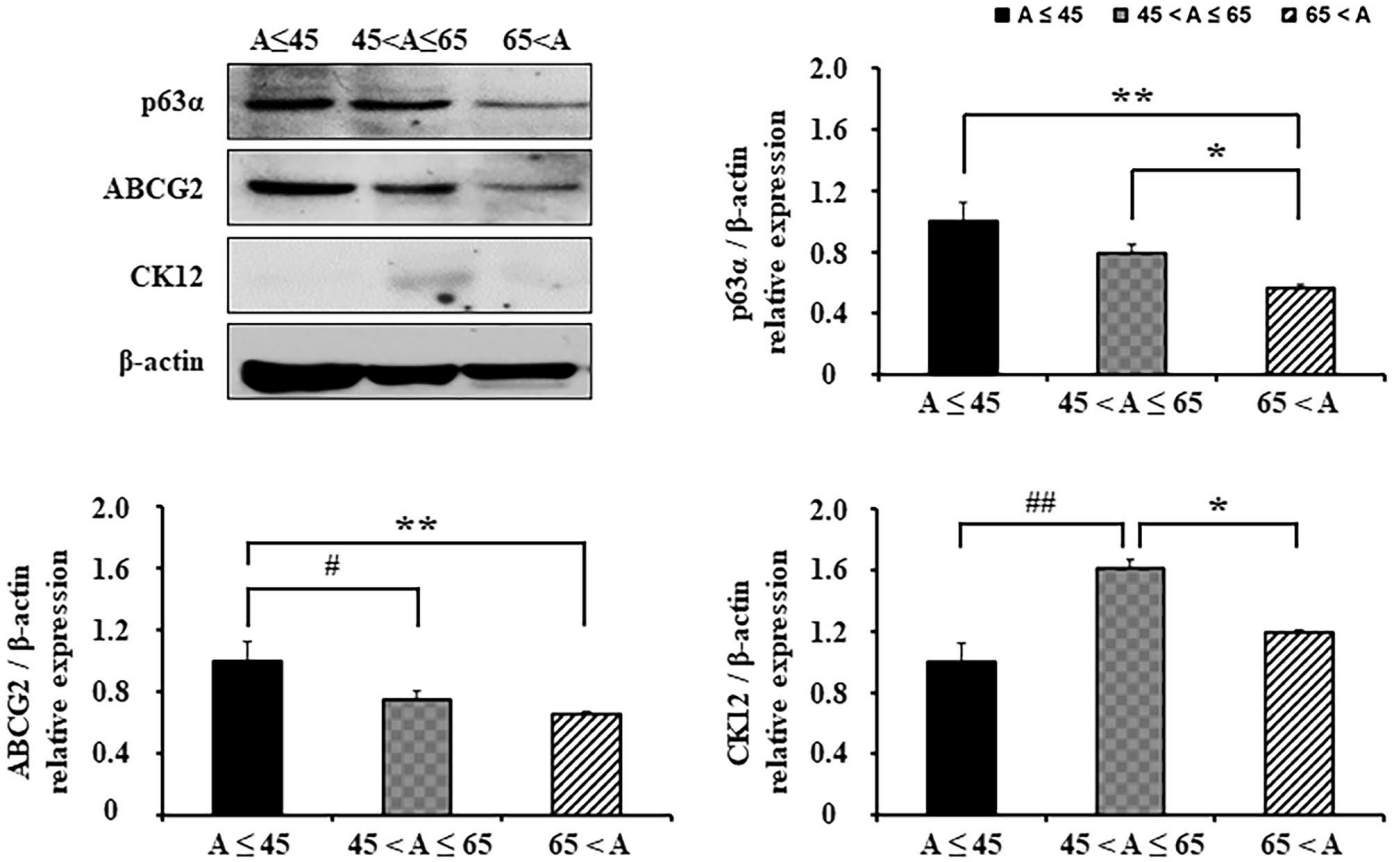
Protein expression of limbal epithelial outgrowth sheets from different donor’s ages. Representative western blot images of p63α, ABCG2 and CK12. The protein expressions of p63α, ABCG2, CK12 and β-actin were developed from the same gel in western blot. All signal intensities were normalized to the signal generated in the same sample by β-actin. Expression levels of p63a, ABCG2, CK12, and β-actin were obtained from the same gel in the western blot. *p < 0.05 (vs. 65 < A), **p < 0.01 (vs. 65 < A), ^#^p < 0.05 (vs. 45 < A ≤ 65), ^##^p < 0.01 (vs. 45 < A ≤ 65), n = 6 from 3 donors.

The protein expression of ABCG2, p63α, and CK12 was confirmed by western blot. Western blot analysis showed that in ABCG2 and p63α, protein expression was significantly higher in the age ≤ 45 group compared to the age > 65 group (p63α; 0.57 ± 0.079, ABCG2; 0.65 ± 0.033, p < 0.01, respectively) (Fig. 3). Relative expressions of p63α in the age 45–65 group was significantly higher than those in the age > 65 group (0.79 ± 0.102, p < 0.05). CK12 expression in the age 45–65 group (1.61 ± 0.161) was revealed to be significantly higher than the other two age groups (p < 0.01, vs. Age ≤ 45; p < 0.05, vs. Age > 65) (Fig. 3).

**Figure 4:**
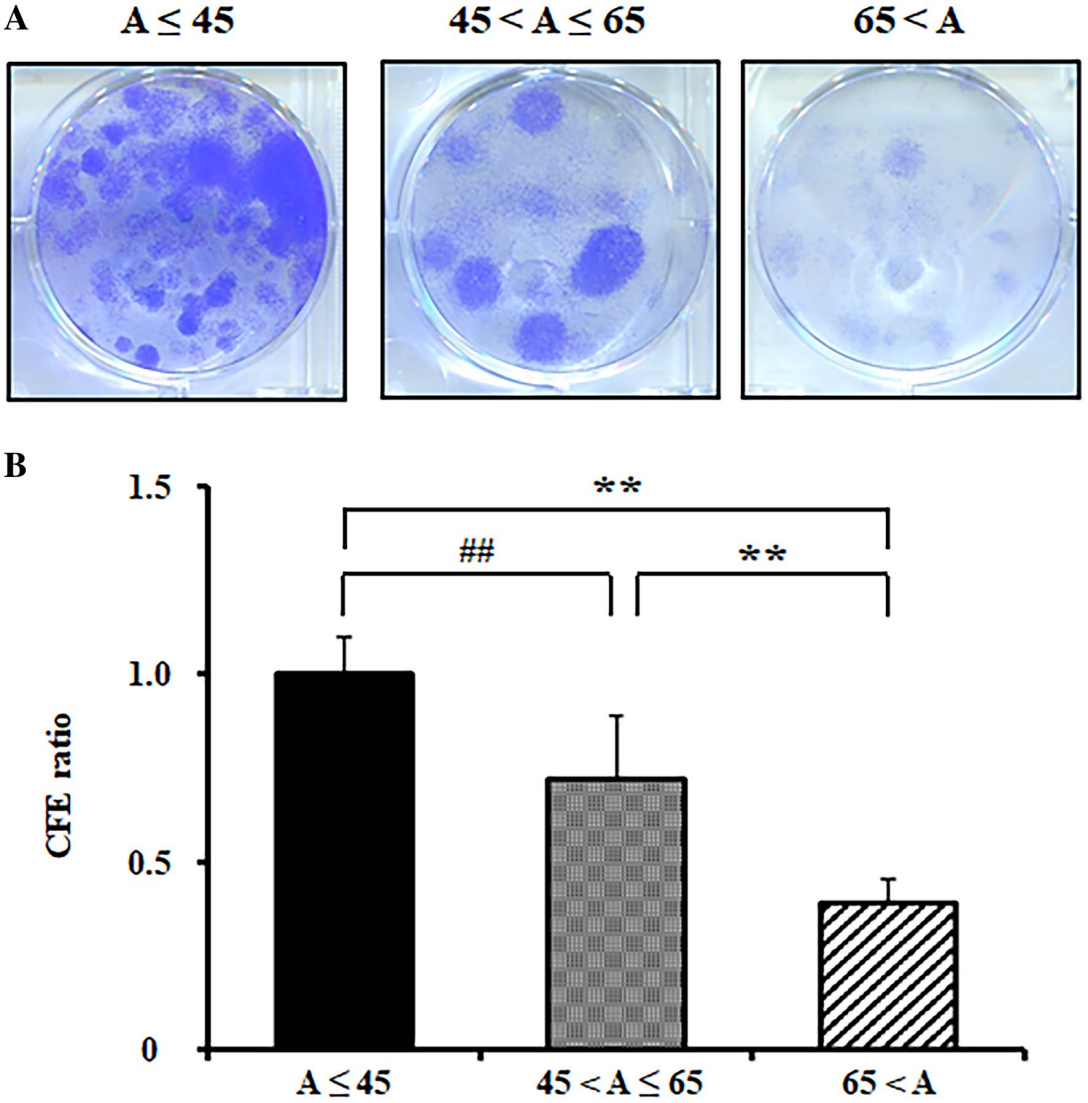
Population clonal index of the limbal epithelial outgrowth sheets from different donor’s ages. (**A**) Coomassie blue stained holoclone colonies from limbal explant outgrowth sheets in CNTP media. (**B**) Colony forming efficiency (CFE) ratio normalized to age ≤ 45. **p < 0.01 (vs. 65 < A), ^##^p < 0.01 (vs. 45 < A ≤ 65), n = 6 from 3 donors.

Finally, we investigated the effect of the donor’s age on the preservation of clonogenic capacity in outgrowth populations. Cells from the age ≤ 45 group showed a higher clonogenic capacity than those from the other two age groups (45 < Age ≤ 65 CFE ratio = 0.7 ± 0.16, p < 0.01; 65 < Age CFE ratio = 0.3 ± 0.06, p < 0.01, vs. Age ≤ 45) (Fig. 4).

### Effects of donor’s age on the activation of limbal stem/progenitor cells in limbal explant outgrowth sheets during the storage period

**Figure 5:**
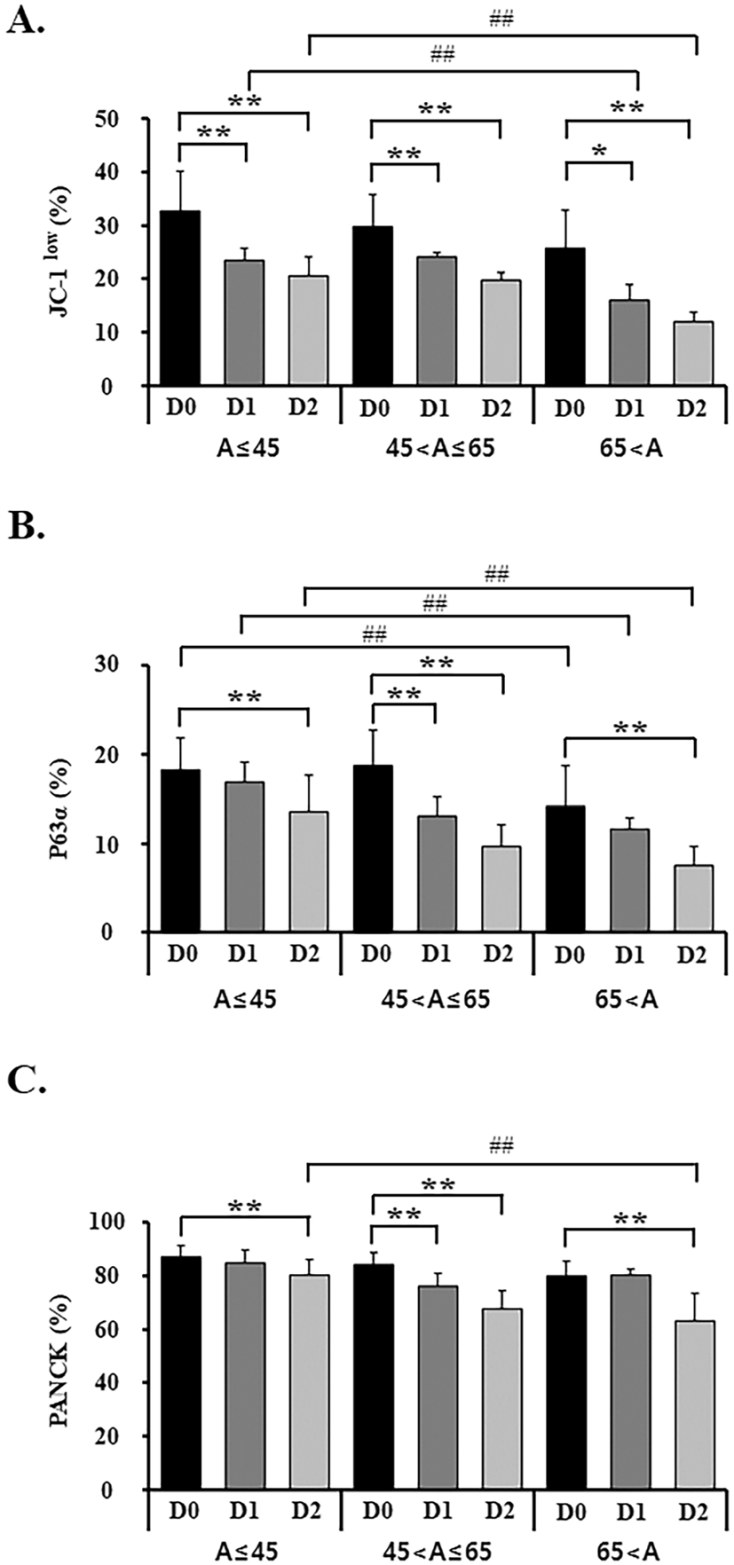
Preservation effects of stem/precursor properties of limbal epithelial outgrowth sheets from different donor’s ages on the storage period. (**A**) Percent of JC-1^low^ cells from FACS analysis and relationship with donor age are shown. JC1 dye exclusion reflects ABCG2 activity and result in the JC1^low^ cohort. (**B**,**C**) p63α and PANCK positive cells from FACS analysis are shown. *p < 0.05, **p < 0.01 (vs. D0, storage period), ^##^p < 0.01 (vs. 65 < A, age), n = 6 from 3 donors.

After the completion of the cell growth from the limbal explant outgrowth sheets, the changes in the characteristics of the cells depending on the age of the donor were observed during the storage period. After the completion of the cell growth, the limbal explant outgrowth sheets were stored in 4 °C for 2 days and analyzed for JC-1, p63α, and PANCK with FACS on each day. All of the cells from the limbal explant outgrowth sheets showed a decrease in the percentages of cells resisting staining by JC-1 depending on the storage period. In the age ≤ 45 group, storage in 4 °C for 2 days resulted in the reduction of the percentages of cells resisting staining by JC-1, as can be seen by comparing Day (D) 0 (32.7 ± 7.50) with D1 (23.4 ± 2.26, p < 0.01) and D2 (20.5 ± 3.57, p < 0.01, respectively) (Fig. 5A). The decreases of JC1 percentage by storage period on the other two groups were observed. In the age > 65 group, storage in 4 °C for 2 days had a significant impact on the decrease in the percentages of cells resisting staining by JC-1 (D1, 16.0 ± 2.80, p < 0.05; D2, 11.9 ± 1.75, p < 0.01), as well as a significant decrease compared with the age ≤ 45 group on D1 and 2 (p < 0.05). The positive cells of p63α on D2 also showed a significant decrease depending on the storage period compared with D0 in all age groups (p < 0.01, respectively). In the age > 65 group, positive cells of p63α on D0, 1, and 2 were significantly lower compared to those in the age ≤ 45 group (p < 0.01, respectively). Additionally, positive cells for PANCK demonstrated significant decrease on D2 of the storage period compared with D0 in all age groups (p < 0.01, respectively) as well as a statistically significant reduction depending on the age group on D2 (Fig. 5).

## Discussion

This study showed that donors younger than age 65 are a better source for limbal epithelial stem cell sheet generation on HAMS with high regeneration potential. Especially, limbal epithelial stem cell sheets from donors in the age ≤ 45 group demonstrated enhanced stemness as well as high proliferation potential.

Our results indicate that limbal explant outgrowth populations on HAMS in the age ≤ 45 group showed accelerated outgrowth compared to those in the age 45–65 and age > 65 groups in the early growth period. Limbal explant outgrowth size in the age ≤ 45 group was significantly larger than the other two age groups on the final growth day of D12. Even though the growth after 9 days is not different in the groups, the expression of stem cell-related markers (p63α and ABCG2) is comparatively higher in the younger age groups. The outgrowth capacity of limbal explants on HAMS was higher than the average outgrowth achieved in the previous studies^20,21^. Our group already demonstrated that limbal epithelial sheets from explant on HAMS showed faster growth, higher CFE, ABCG2 efflux activity (JC-1^low^ cells), and p63α expression compared to sheets on transwell only^16^. Thus far the application of HAM as a substrate in clinical settings included HAM on the transwell insert^12,22–24^ or inter-lockable plastic amnion rings^15,25^. In our study, we designed HAMS in which HAM was spread and tucked around a glass slide tightly fit in a 35 mm^2^ dish. This HAM slide scaffold provided a smooth surface and mechanical tension for HAM, resulting in fast growth of limbal explant outgrowth sheets and higher cell yield compared to HAM on transwell. Our limbal outgrowth cells on HAMS constituted more than 15% p63+ cells of the total cells by FACS analysis, which was significantly higher compared to cells on transwell and a previous study by Rama et al.^8,16^. Kethiri et al. demonstrated that explants as small as 0.3 mm^2^ for live and ≥ 0.5 mm^2^ for cadaveric tissue obtained an in vitro mean outgrowth area of 182 mm^2^ and 218 mm^2^ which was lower than those in our study^20^. In addition, Utheim et al., reported that the 3 mm-sized limbal explants and 1 mm-sized limbal explants yielded the mean explant outgrowth area as 68.6 mm^2^ and 48.5 mm^2^, respectively^21^.

Donor characteristics have been proven to influence the growth capacity of limbal epithelial stem cells. For instance, a previous study by Utheim et al. suggested that limbal explants obtained from the superior limbus demonstrated the highest outgrowth capacity^26^. In addition, Utheim et al. showed that small explants have higher growth potential and grow faster than larger explants^21^. Our study showed that the limbal explant outgrowth sheets from young donors preserved a significant number of effective limbal stem/progenitor cells. FACS analysis indicated significantly more p63α^+^ cells in outgrowth cells in the age ≤ 45 group compared to the age > 65 group but no significant difference from those in the age 45–65 group. The percentage of JC-1^low^ cells, which represents limbal stem/progenitor cells, showed significant negative correlations with individual donor age. CFE decreased significantly according to the donor age as well. Ki67-positive cells were significantly lower in the age > 65 group than the other two age groups. In a previous study by Notara et al., levels of putative stem cell markers and telomerase activity did not differ according to donor age but CFE decreased significantly with increasing donor age^18^. These findings are in line with our study.

However, the study with limbal stem cell cultures by Nieto-Nicolau et al. showed that the proportion of p63+ cells, a putative stem cell marker, was 3.9%, 18.12%, and 16.75% in the donor groups aged < 60 years, 60–75 years, and > 75 years, respectively, suggesting that LSC cultures from aged donors can express ≥ 3% of p63+ cells—considered as the minimum value for predicting favorable clinical outcomes after LSCT^27^. The authors suggested that the lower percentage of p63+ cells in the donor age < 60 years appears to be from the inter donor variation in the study, and the relatively small number of donors under the age of 60 may be the cause^27^.

To determine the clinical usefulness of this current study, we investigated the impact storage period had on the limbal explant outgrowth sheets for different age groups. Collectively, in observing the effect the variations of storage period had on the changes for JC-1^low^ cells, positive cells for p63α and PANCK, the older age group exhibited a significant reduction of these cell differences on Day 2 compared to D0. According to a previous study comparing the stem cell activity at 0, 1, 4, and 7 days at 4 °C, the stem cell activity decreased significantly on the 4th day of storage^28^. These findings demonstrated that donor age might play an important role in the developing of the limbal epithelial stem cell sheet, as well as the maintenance of stemness during storage for clinical applications.

The strength of this study is that the younger donor age is more advantageous for limbal epithelial stem cell sheet generation on HAMS developed by the authors. It was confirmed that the outgrowth capacity of limbal explants on HAMS was higher than the average outgrowth of previous studies^20,21^. Also, by examining how the storage period changes according to donor age, it can be expected to serve as a guideline that can be used when clinically applied to patients.

In conclusion, our study suggests that younger donors, especially those of age < 65, are a better source for allogenic limbal epithelial stem cell sheet generation with high regeneration potential and maintenance of stemness.

## Materials and methods

### Tissue procurement

Post-keratoplasty discards of human corneal-limbal tissues from unidentifiable cadavers were obtained from Seoul St. Mary’s Hospital Eye Bank (Seoul, Korea). The Institutional Review Board determined that the use of these tissues did not constitute research on human subjects. All methods were performed in accordance with the relevant guidelines and regulations. Informed consent was obtained from all subjects and/or their legal guardian(s). Tissue acceptance criteria included (1) tissue harvest occurring within 12 h of death; (2) tissue storage in Optisol (Bausch & Lomb, Rochester, NY) for less than 72 h after harvest, and (3) donor testing negative for human immunodeficiency virus, hepatitis B or C, Epstein-Barr virus, and syphilis. Written informed consent was obtained from women before cesarean-section delivery when human amniotic membrane (HAM) was obtained and its use was approved by the Institutional Review Board of the College of Medicine, The Catholic University of Korea. Under sterile conditions, HAM was washed, placed over a nitrocellulose membrane, and preserved in tissue culture medium and glycerol at a ratio of 1:1 and stored at − 80 °C until further use. Post-keratoplasty cornea rims in Optisol were split into 12 equal parts in the laboratory. Two-thirds of the bottom sclera tissues were trimmed and were cut into limbal strips about 0.5-mm-wide each. The limbal strips were not stored, but always directly deposited onto the human amniotic membrane slide scaffold (HAMS) right after they were cut.

### Culture medium

Supplemented hormonal epithelial medium (SHEM) is composed of 950 ml of a 1:1 mix of Dulbecco’s modified minimal essential medium and HAM F12 (DMEM/F-12, Gibco, Grand Island, NY), 50 ml of human AB serum (Sigma), 0.5% dimethyl sulfoxide (Sigma), 5 µg of human recombinant epidermal growth factor (Sigma), 14 mg of O-phosphoethanolamine (TCI, Tokyo, Japan), 5 mg of ethanolamine (Sigma), 1 × insulin-transferrin-selenium (Gibco), and 1 × penicillin–streptomycin (Gibco) mixes.

### Limbal epithelial stem cell sheet generation from human limbal explant outgrowth culture on human amniotic membrane

After thawing, HAM was treated with cold 5 M urea (Sigma) for 5 min at room temperature and scraped gently with a #15 blade to remove remaining epithelial cells. De-epithelialized HAM was transferred onto the surface of a slide glass (26 × 26 mm) with the epithelial side facing upward so it enveloped the four corners of the slide glass, ensuring there were no wrinkles. The HAMS was tucked into a 35-mm culture dish and pre-equilibrated overnight in culture media. Limbal strips were deposited, epithelial side up, and cultured in air–liquid interface conditions (Fig. 1A)^16^. Every 72 h, culture media were refreshed with enough medium so as to barely cover the explant exposed surface. Our previous study demonstrated limbal epithelial outgrowth sheets on HAMS consisted of 3–6 cell layers with attached to the HAM on transmission electron microscopy analysis^16^. On day 12, the limbal explant outgrowth sheets were treated with Dispase II (2 mg/ml; Roche, Indianapolis, IN) overnight at 4 °C. The outgrown cells were incubated in TrypLE (Gibco) for 10 min to obtain fully dissociated cells.

### Immunofluorescence

Limbal explant outgrowth epithelial sheets were fixed with cold methanol for 10 min, permeabilized with 0.1% Triton X-100 in PBS for 30 min and incubated with 10% goat serum for 1 h to block nonspecific reactions. Cells were then incubated with anti-Ki67 (mouse monoclonal, Santa Cruz), washed with TBS twice, and incubated with Alexa Fluor 488-conjugated anti-mouse IgG Ab. Stains were captured by confocal microscopy in an LSM 510 Meta confocal microscope (Carl Zeiss, Oberkochen, Germany). The Ki67 stains were quantitated in Photoshop software (Adobe Systems, Santa Clara, CA). The luminosity (i.e., stain intensity) of the background areas (range, 0–255; 8 bit luminosity scale) was determined with the Photoshop histogram function. We counted the number of pixels with luminosity equal to or above the luminosity of the highest 1% of the background range pixels. Blue (DAPI+) luminosity is assumed to be proportional to the number of nuclei present in the field and hence a relative measure of nuclear count. Alexa 488 luminosity is assumed to be proportional to the number of antigen units present in these nuclei and hence the Alexa 488/DAPI ratio is proportional to the density of Ki67 epitopes per nuclei. Three randomly selected areas of equal size and sufficiently large to incorporate at least 100 nuclei were counted to obtain mean ± SD for each condition tested. For graphic representation, all values were normalized by the control value, which was set as equal to one.

### Flow cytometry

For ABCG2 efflux activity, a property tightly linked to multiple somatic stem cells including the limbal stem cells^29,30^, 2 × 10^4^ cells from limbal explant outgrowth sheets were seeded overnight in SHEM. They were incubated for 45 min with 250 nM JC1 (Axxora, San Diego, CA), released by trypsinization and diluted in FACS buffer. JC1, a mitochondrial binding dye displaying an accumulation-dependent bathochromic emission shift, is an ABCG2 substratum. In cells displaying high ABCG2, its efflux activity prevents JC1 from reaching its mitochondrial binding sites. Thus, in flow cytometry bivariate 531(green)/585(orange) emission plots, these cells appear as a low-stain cohort (JC1^low^) lying on the left of the cohort of low/nil ABCG2 fully stained cells^19^. Studies were performed by a FACS Calibur (BD Biosciences, San Diego, CA) instrument.

To stain for p63α (Cell Signaling Technology, Beverly, MA) and Pancytokeratin (PANCK; Abcam, Cambridge, MA), cells were (a) enzymatically harvested; (b) fixed with 10% formalin for 10 min; (c) permeabilized with 0.1% Triton X-100; (d) incubated with 5% BSA for 30 min; (e) incubated with a rabbit polyclonal antibody recognizing p63α and a mouse monoclonal antibody recognizing PANCK for 30 min; (f) incubated with Alexa-488-conjugated goat anti-rabbit and goat anti-mouse IgG (Thermo Fischer, Waltham, MA) for 30 min; and (g) suspended in FACS buffer^16^.

### Western blot

Cells from limbal explant outgrowth sheets were lysed with lysis buffer containing phosphatase inhibitor cocktail 2 (Sigma) and protease inhibitor cocktail (Roche). Equal amounts of protein in cell lysates were separated by 10% SDS-polyacrylamide gel electrophoresis under reducing conditions and electro-transferred to a PVDF membrane (Millipore, Billerica, MA). The membrane was blocked by 5% skim milk and incubated at 4 °C for 18 h with a mouse monoclonal antibody recognizing ABCG2 (Abcam), or a rabbit polyclonal antibody recognizing p63α, or a goat polyclonal antibody recognizing cytokeratin 12 (CK12, Santa Cruz Biotechnology, Santa Cruz, CA). The membranes were washed three times in TBST and incubated at room temperature for 1 h with the appropriate secondary antibodies conjugated to horseradish peroxidase. After three washes of the membrane, protein bands were detected using an enhanced chemiluminescence reagent (ECL; Amersham Biosciences, Piscataway, NJ). All membranes were stripped and reprobed with mouse monoclonal anti-β-actin antibody to provide a normalizing reference. We performed four to six independent experiments and calculated relative levels of expression by image analysis.

### Clonal proliferation

Cells harvested from limbal epithelial sheets by trypsinization were seeded on collagen type I (PureColl^tm^; Biomatrix, San Diego, CA)-coated 6-well plates at a rate of 100 cells/cm^2^ in CNTP medium (Cell-N-Tec; Bern, Switzerland). CNTP was previously shown to preserve proliferative capacity, colony-forming efficiency, and stem cell-like phenotypes of human corneal epithelial cells^31^. Colony formation was monitored daily and analyzed on day 14 after fixation in cold methanol and staining with 0.45% Coomassie blue R 250 dissolved in 10% acetic acid–45% methanol.

### Statistical analysis

Statistical significance between groups was examined by a nonparametric, two-tailed Mann–Whitney *t* test using SPSS 14.0 version. We regarded p < 0.05 as significant, p < 0.01 as highly significant, and p < 0.001 as extremely highly significant.
